# Spanish Consumer Purchase Behaviour and Stated Preferences for Yoghurts with Nutritional and Health Claims

**DOI:** 10.3390/nu11112742

**Published:** 2019-11-12

**Authors:** Petjon Ballco, Tiziana De Magistris

**Affiliations:** 1 Unidad de Economía Agroalimentaria y de los Recursos Naturales, Centro de Investigación y Tecnología Agroalimentaria de Aragón (CITA), Gobierno de Aragón, 50004 Zaragoza, Spain; petjonballco@gmail.com; 2 Instituto Agroalimentario de Aragón, IA2 (CITA-Universidad de Zaragoza), 50009 Zaragoza, Spain

**Keywords:** choice paradigm, nutritional claims, health claims, heterogeneous preferences, Spain

## Abstract

Nutritional and health claims are a useful tool for promoting healthier food choices and prevent non-communicable disease[s] (NCDs). Exhaustive literature that has investigated consumer evaluation of the presence of nutritional and/or health claim(s) during the decision-making process suggests that consumers’ sensitivity towards nutritional claims (NCs) and health claims (HCs) are still fragmented and should be further investigated. Our objective is to study the relationship between choice behaviour, attitudes and socio-demographic characteristics in order to evaluate the effectiveness of consumer characteristics in predicting Spanish consumers’ choice of products with NCs and HCs. A discrete choice experiment for yoghurt was conducted on a sample of 218 Spanish consumers, stratified by age, gender, education level, and income. Applying a latent class approach has enabled us to identify a niche of individuals, sensitive of NCs and HCs and to characterize them with respect to the rest of population. Results suggest that consumers positively valued most claims, however, the valuation was heterogeneous, and three consumer segments were identified: ‘health-claims oriented’, ‘nutritional- and health-claim oriented’ and ‘indifferent’. The results supply insights for the development of more targeted promotion campaigns, as well as for further actions in food marketing.

## 1. Introduction

The epidemic of overweight and obese individuals presents a major challenge to chronic-disease prevention and to health over the course of life worldwide. Fuelled by increasingly sedentary lifestyles and a nutritional transition towards processed foods and high-calorie diets, many countries have witnessed the prevalence of obesity amongst its citizens double, even triple [1]. One key mechanism that policymakers have presented to encourage healthier eating is the provision of information on food packages via nutritional labels [2], such as nutritional claims (NCs) and health claims (HCs) [3]. Both types of claims are an attempt by the European Union (EU) Regulation (EC) No. 1924/2006, with the aim to help consumers make well-informed choices [4,5] at a glance [6]. However, NCs and HCs are credence attributes. This type of attributes is neither directly observable by consumers before purchase, nor can it be experienced after purchase [7,8]. Therefore, to guarantee trustworthy information to consumers the European Food Safety Authority (EFSA) requires that NCs and HCs in food products be based only on scientific evidence [9]. Since the introduction of the EU regulations, the agro-food industry has increasingly made efforts in the innovation processes to obtain healthier products by reducing saturated fats, sugars, and salt, while the retail sector has increased considerably the presence of processed products with NCs and HCs in the EU markets. In 2015 about 85% of all packaged food products in Europe were sold with NCs [10,11] with Spain ranking as second, after the UK [10]. Regarding the type of claims used in the Spanish market, Cuevas (2012) reported that the NCs with the highest presence pertain to food products that are: rich in fibre (47.5%), without added sugar (41%), free of saturated fat (41%), low in calories (39%), rich in whole grains (34%), rich in vitamins and minerals (26%), low in salt or salt-free (25%), and rich in omega-3 fatty acids (22%) [12]. Similarly, Royo-Bordonada et al. (2016) who examined the availability of food with NCs and HCs in Spanish television advertisements over a seven-day period identified 169 food products, of which 28.5% belong to the dairy group and 60.9% to the non-core or miscellaneous category. A total of 53.3% of products contain NCs, and 26.6% contain HCs. Low-fat dairy products are the category with the highest percentage of NCs and HCs [13]. Finally, a more recent study by Lopez-Gálan and de-Magistris (2017) on the presence of NCs in the Spanish market found that, out of 4568 product types, about 900 contain NCs. The most frequent nutrients found are related to the fat (42%), sugar (32%), dietary fibre (20%), and salt (6%) contents. The results from these studies demonstrate that Spanish consumers have access to food alternatives with NCs and HCs, however it has been reported that only a very small percentage of consumers purchase them [14]. 

Beside the availability and exposure to the market of foods with NCs and HCs, other factors that affect the purchase of food with these claims are several attitudinal and cognitive characteristics, which are related to nutritional and health knowledge, understanding, interest in healthy eating, and socio-demographic characteristics (see [15,16] for an overview). Understanding the NCs and HCs provided on the FOP implies that consumers recognise and know what each nutrient term and measurement unit means. It also assumes that they understand the relationships between the different nutrients and the role of each nutrient in the body [17]. In this regard, Prieto-Castillo et al. (2015) report that over half of the participants in Madrid (52.4%) stated to have a full understanding of nutrition labels. The highest percentage was found in consumers over 65 years old (63.6%), retired (62.5%), living alone (62.1%), and with a high level of education (61.8%). Higher education was also found to be positively correlated with information search and self-perceived understanding of NCs in another Spanish study [11]. Regarding knowledge towards foods with nutrition labels, previous research noted that consumers’ knowledge of the nutritional properties of food products play a role in the importance associated with the labelled claims, as it may increase the perceived benefits of the product [18,19]. Two Spanish studies [20,21] indicated that a higher level of nutritional knowledge is linked to healthy individuals, with high income, and households with children who are more motivated to search for nutrition information. Hence, Spanish consumers with greater knowledge of nutrition information are more likely to use nutritional labels [21]. Finally, the need for information about food, diet and health is driven by most importantly, consumers’ use and interest in healthy eating [22]. One may have sufficient knowledge of the nutritional properties of the food product and understand the labels, but not the interest in healthy eating and use of NCs and HCs in the decision-making. Hence, consumers’ use and interest in healthy eating is the attitudinal characteristic studied in this research as these type of consumers tend to be more engaged in health-promoting behaviours [23]. 

In overall, products with NCs and HCs have been considered to be part of a healthy diet [23], and the appeal of HCs is positively linked to the interest in healthy eating [24]. However, research regarding preferences and interest in healthy eating of food with NCs and HCs in Spain is limited and the results are mixed. Specifically, Barreiro-Hurle et al. (2010) report that although individuals use nutrition-facts panels and NCs, most consumers use only one of these claims (33%) and of these, the majority pay no attention and show a low interest in using NCs (68%) [20]. This is also consistent with the results of Prieto-Castillo et al. (2015), who found that only a small percentage of individuals in Spain were interested to use NCs [11]. Lastly, López-Galán and de-Magistris (2019) who explored the effects of emotional eating in the purchase behaviour, found that emotional eating had a negative impact on the purchase behaviour of food with NCs [25]. On the contrary, recent research on consumer preferences for NCs and HCs in Spain suggest that preferences are heterogeneous. In particular, de-Magistris et al. (2016) assessed the influence of body image on consumer preferences for potato chips carrying NCs among obese and normal-weight participants. Their findings indicated that obese people with body-image dissatisfaction were willing to pay more for healthier chips compared to normal-weight participants with the same problem [26]. Finally, Jurado and Gracia (2017) examined Spanish consumer evaluation of NCs (i.e., high in fibre and reduced saturated fat) on breakfast biscuits. They report that consumers positively valued both NCs, and premium prices may be attached to targeting either of two subpopulation segments (low-saturated-fat seekers and high-fibre seekers) [27]. In our view, these studies are important. Nevertheless, we believe that the full advantage of using multiple types of NCs and HCs was not taken. In overall, the results from this literature suggest that our understanding of Spanish consumers’ sensitivity towards NCs and HCs is still fragmented and should be further investigated. 

Given the aforementioned, the purpose of this research is to examine the relationship between choice behaviour, attitudes, and socio-demographic characteristics, and evaluate the effectiveness of consumer characteristics in predicting Spanish consumers’ choice of products with NCs and HCs. To achieve these objectives, we used a discrete choice experiment (DCE) on plain yoghurts. To find out whether there is a segment of Spanish consumers responsive to NCs and HCs and how it differs from the rest of population, we applied the latent class (LC) approach which permits an analysis of determinants of consumer choices, taking into account the heterogeneity that may exist between different segments. 

This study focuses on NCs and HCs because they are a simpler way of presenting information compared to nutritional tables. They do not list the amount of a nutrient, but instead summarise the information for a specific nutrient and communicate it to consumers in simple, easy-to-process language (e.g., fat-free). We chose yoghurt as a product of reference, as it has been recommended as part of a healthy diet in many countries [28], and it contains the most NCs and HCs among all the food products in Spain (From a market analysis on various food products present in different hypermarkets and supermarkets in Spain, it is the product that carries the most NCs and HCs). We chose Spain as the location of research due to the high number of NCs and HCs available in the Spanish market [10,27]. While the existing literature provides a wealth of insights into attitudinal and cognitive characteristics such as nutritional and health knowledge, and understanding of food products with NCs and HCs, to the best of our knowledge, this is the first study that analyses consumer heterogeneity in preferences for multiple NCs and HCs on the front of pack (FOP) by identifying Spanish consumer segments. The characterisation of consumers based on categories would allow food companies and public authorities to tailor strategies to promote healthy food choices. 

## 2. Materials and Methods 

### 2.1. Discrete Choice Experiment: Product and Attribute Selection 

It is worth mentioning that an NC indicates only the nutrient on the FOP of the yoghurt, while an HC presents both the nutrient (i.e., NC) and a description of its health benefits. The selection of NCs and HCs used in this study was conducted following the official definitions from the EU regulations (EC) No. 1924/2006. To determine their presence in the market, we created a database that collects information regarding food products with both types of claims available in the Spanish market between July and September 2015. The products included in the database were selected based on their importance in the shopping basket of Spanish households (According to the Ministry of Agriculture and Fisheries, Food and Environment—MAPAMA, (2014) Consumer Observatory in Spain, 89% of the per-capita consumption of packaged food consists of liquid milk, processed meats, yoghurts, cheeses, and industrial bread and biscuits) [29]. From the results of this database, we chose yoghurt for further analysis, because it carries the most NCs and HCs, is considered a healthy food product and is frequently consumed by Spanish households [29]. In total, 251 yoghurts that carry one NC and 67 with one HC on the FOP correspond to the official EU definitions (Regulation (EC) No. 1924/2006 [3]. All the products used are plain yoghurts with no added flavours or fruits, except for one added-fibre variety, which contains several types of cereals (oats, barley, wheat, and wheat bran). An unlabelled yoghurt was also selected as the baseline for comparison. Table 1 presents the NCs and HCs that were presented to consumers. Previous research suggests that, overall, HCs are not fully understood by the ‘average consumer’ (EU Regulation 1924/2006 Recital 15 defines the average consumer as someone ‘who is a reasonably well informed and reasonably informed observant and circumspect, taking into account social, cultural and linguistic factors’) [30,31]. Hence, in addition to the ones present in the local market (numbers 3, 5, 7, as reported in Table 2), we extracted five additional HCs from Regulation (EC) No. 1924/2006 (numbers 1, 2, 4, 6, 8 in Table 2) that are easier to understand, according to a focus group of 20 ‘average consumers’ of different ages and education levels surveyed before the experiment. Based on the market database, we selected a 500-g package (four containers, each with a weight of 125 g), because it is the most common size on the market. 

Concerning the price, two Spanish studies found that consumers who pay more attention to price when shopping are less likely to use NCs and HCs [20,21]. Therefore, our study followed the methodology of Carlsson et al. (2007) who conducted a DCE without the price attribute [32]. Other investigations that exclude price were performed by Bialkova and van Trijp (2011) [33] and Bialkova et al. (2014) [34]. As with Carlsson et al. (2007), we told the participants that all the options cost the same amount, since yoghurt is regularly consumed in Spanish households (According to the results from the Consumer Observatory in Spain (Ministry of Agriculture and Fisheries, Food, and Environment) [29] and the questionnaire on consumption frequency, 56% of households consume yoghurt once a week, and 14% do so twice a week), and the individuals are aware of the price variations (The yoghurt market prices in October 2016, for a 4 × 125 g pack, were: natural (€1.09), fat-free (€1.80), low in sugar (€1.92), source of fibre (€1.99), source of vitamin B6 (€1.99), and source of calcium (€1.69)), for different types of yoghurts.

**Figure 1:**
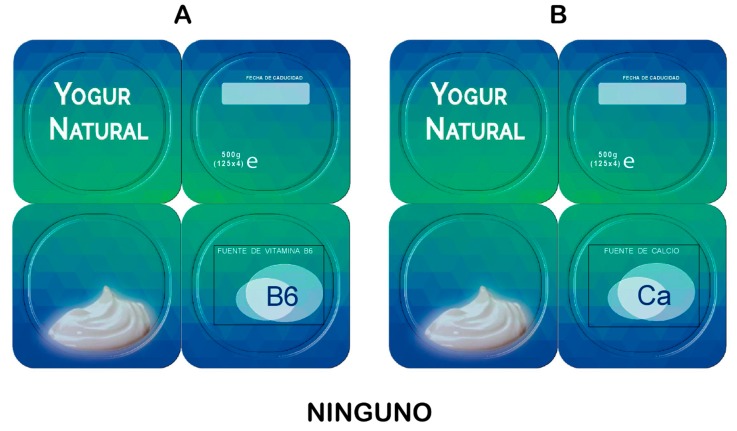
An example choice task. Option A represents the Spanish version of yoghurt with a *source of vitamin B_6_*, and option B refers to one with a source of calcium. ‘*Ninguno*’ is the ‘no-buy’ option.

**Figure A1:**
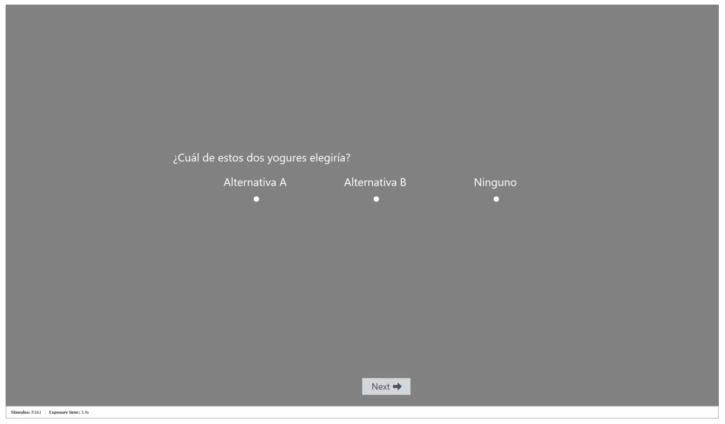
An evaluation form of choosing the most preferred yoghurt. Note: The question is translated from Spanish: Which of these two yoghurts would you choose? “Alternativa A‘ refers to option A, ’Alternativa B‘ refers to option B and ’Ninguno‘ is the ’no-buy’ option.

Using the NCs and HCs listed in Table 1 and following the experimental design employed by Bialkova and Trijp (2011) and Bialkova et al. (2014), we applied an availability design [35]. The experimental set-up resulted in 91 possible choice tasks or questions, excluding repeated ones (mirror-effect choice questions). To reduce this number and prevent fatigue effects, we only used 44 choice questions (According to the main objective of the study, the 44 choice questions included all the product alternatives combining NCs and HCs), which were randomly split into four blocks of 11 choice tasks for each participant. The respondents were then randomly assigned to only one of the blocks, thus, each person only answered 11 choice questions, which were also presented in random order. Each question is composed of three alternatives: two yoghurts, each with a different HC and NC level, and a no-buy option (see Figure 1). The DCE was presented on a computer screen. After observing the two product combinations, the participants selected their preferred one on an evaluation form (see Appendix A, Figure A1 for an example of the evaluation page) presented after each choice task. 

### 2.2. Participants and Recruitment

The experiment was conducted in 2016 in Zaragoza, Spain, which is popular among food marketers and consulting companies since the socio-demographics of the town are representative of the Spanish Census of Population (see Appendix A—Table A1). (This study is part of a larger investigation of consumer behaviour regarding NCs and HCs in Spain, where multiple experiments have been conducted) For the selection of participants, an external company recruited individuals who consumed yoghurt, were responsible for the food purchase in the household, and were older than 18 years at the time of the study. 

#### Implementation Procedure and Measures

Upon arrival, participants received information on the main purpose of the experiment and signed a document to indicate their informed consent. An ID number was assigned to each respondent to guarantee anonymity. Subsequently, a general overview of the whole working session and the approximate duration was provided. Consumer choices were measured by asking the respondents to make 11 selections between two products with different NCs and HCs and a no-buy option. They were reminded throughout the session to imagine that they were in supermarket purchasing yoghurt for their regular consumption. 

After choosing their preferred yoghurt with NCs and HCs, the participants completed a brief questionnaire. The first part of the questionnaire measures purchases and consumption frequency. Besides, the respondents were asked to rate the importance to which they attach different attributes when purchasing yoghurts on a 5-point scale. The second part assesses knowledge associated with various nutrients and substances and the recommendations of health experts (see [36] for an overview). The third part of the questionnaire measures the use of nutritional information (i.e., whether the participants pay attention to NCs and HCs on the products they buy) on a 4-item and a 5-point Likert scale (e.g., ‘I use the nutritional information on the label when making most of my food selections’). The response options range from ‘completely disagree’ (1) to ‘completely agree’ (5), with a Cronbach’s α of 0.69. Interest in healthy eating was evaluated on an 8-item and a 5-point Likert scale (e.g., ‘It is very important to me that my diet is low in fat’), with options ranging from ‘completely disagree’ (1) to ‘completely agree’ (5) and a Cronbach’s α of 0.76 (see [37] for an overview). Lastly, the participants were asked to report their socio-demographic consumer characteristics (e.g., gender, family size and composition, age, educational level, and income bracket). Cross-tabulations with χ^2^ statistics were used to test for any association between the categorical variables. For the comparison of mean scores, we used the Kruskal–Wallis rank test instead of the Anova-Bonferroni, because the results from the Shapiro-Wilk test demonstrated that our data are not normally distributed.

### 2.3. Model Specification and Estimation 

Our theoretical model is based on the Lancastrian consumer theory of utility maximisation [38]. Lancaster (1966) proposes that the total utility associated with the provision of a good can be decomposed into separate utilities for theoretic component attributes. However, this utility is known to the individual and not to the researcher. The researcher observes some attributes of the alternatives, but some components of individual utility are unobservable and hence treated as stochastic (following random utility theory). Therefore, the utility is taken as a random variable, where utility from the *n*th individual facing a choice among *j* alternatives within choice set *J* on the *t*th choice occasion can be represented as:

(1) $$U_{njt}\;=\;\beta X_{njt}+{\;\varepsilon }_{njt}$$

In the above formula, β is the estimated vector of parameters, and $\varepsilon_{njt}$ is an independent identically distributed (i.i.d.) error term over time, individuals, and alternatives. Traditionally, consumers have been assumed to be homogeneous in terms of taste, and conditional logit models have been used [39]. However, numerous choice-experiment empirical studies have found consumer preferences for food products to be heterogeneous, and the specified model needs to allow for variations in the taste parameters of the observed variables in the population. Two alternative models have gained popularity in choice-modelling literature when addressing the issue of heterogeneity: random parameter logit (RPL) and latent class (LC) logit. Both are versions of the mixed logit model [40]. 

The RPL model has been widely used in applications of discrete choice modelling across disciplines, especially in agro-food research [26,27,41,42,43,44,45,46,47]. Heterogeneity is incorporated into this approach via consideration for each individual’s unique set of preferences and estimates of the utility function. When estimating the choice model, an additional vector of parameters is included to incorporate individual preference deviations with respect to the mean values. (*β* in (1) is not constant, but varies across individuals as a variable *βn*) However, if preferences are assumed not to be ‘unique’ for each individual but rather distinct for a set number of individual classes or segments (as referred from this point), the LC model is more appropriate for modelling choices. In this approach, consumers are assumed to belong to different segments, each characterised by different segment-specific utility parameters. In other words, within each segment, consumer preferences are homogeneous, but they vary between segments, reflecting a ‘lumpy’ spread preference and allowing a more in-depth understanding of heterogeneity [40]. This approach has also been used to analyse consumer preferences for agricultural products, enabling the identification of distinct patterns of valuation and behaviour, [13,35,48,49,50,51], among others. In the LC model, the utility of the individual *n* choosing alternative *j* in the *t*th choice alternative is calculated as follows:

(2) $$U_{njt|S\;}\;=\;\beta_{S}X_{njt}+{\;\varepsilon }_{njt|S}$$

where $\beta_{S}$ is a parameter vector of class *S* associated with the vector of explanatory variables, and $X_{njt}$ and $\varepsilon_{njt|S}$ are error terms that follow a Type-I (or Gumbel) distribution. The deterministic proportion of utility can be separated into two components, one related to the choice attributes and another latent one associated with the socioeconomic and psychometric characteristics of the individual [52]. Thus, the probability that an individual will select alternative *i*, conditional on belonging to segment *S*, can be expressed as follows:

(3) $$P_{ni}\;=\;\overset{S}{\underset{S\;=\;1}{\sum }}P_{nS}\overset{T}{\underset{t\;=\;1}{\prod }}P_{njt|S}$$

where $P_{nS}$ is the assignment of individual *n* to segment *S* (i.e., probability of segment *S*), and $P_{njt|S}$ is the probability that individual *n*, conditional on belonging to segment *S* (*S* = 1, …, *S*), chooses alternative *j* from a particular set *J* comprised of *j* alternatives, on choice occasion *t* [53].

The parameters for the attributes and individual characteristics are simultaneously estimated by maximising the likelihood function in the state of incomplete prior information on segment membership or choice probabilities [54]. Subsequently, the number of segments is endogenously determined along with the utility coefficients. The LC model was estimated using NLogit 6.0. Econometric Software, Inc. (http://limdep.com/products/nlogit/). In the LC model, two groups of variables require further specification: those that enter the utility function and those that explain the segment-allocation function. The utility function comprises the attributes analysed, and one alternative-specific constant is given in the following way:

(4) $$\begin{array}{ll} U_{njt}\;=\;\beta_{0\;}nobuy & +\;\beta_{1\;}ncfat_{njt}+\beta_{2\;}hcafat_{njt}+\beta_{3\;}ncsug_{njt}+\beta_{4}hcasug_{njt}\\ & +\beta_{5}ncfib_{njt}+\beta_{6}hcpfib_{njt}+\beta_{7}hcafib_{njt}+\beta_{8}ncvit_{njt}\\ & +\;\beta_{9}hcpvit_{njt}+\beta_{10}hcavit_{njt}+\beta_{11}nccal_{njt}+\beta_{12}hcpcal_{njt}\\ & +\beta_{13}hcacal_{njt}+{\;\varepsilon }_{njt} \end{array}$$

In the above equation, *n* is the number of respondents, *j* represents the available choices in the choice sets (two experimentally designed yoghurt profiles and the no-buy option), and *t* is the number of choice situations. OptOut is the alternative-specific constant representing the no-buy option. The other 13 attributes (as reported in Table 1) enter the model as dummy variables, where the ‘unlabelled’ yoghurt represents the baseline.

## 3. Results

### 3.1. Socio-demographic Characteristics 

Considering the main components of the model discussed in the previous section, we first present the individual differences across the three segments. Participants were selected through random stratification with proportional distribution of age, gender, and education to avoid under-/over-representation of consumer profiles. The final sample consists of 218 individuals. Table 2 shows their socio-demographic characteristics.

Most of the respondents are female (52.8%). The average age of our sample is 49 years. Approximately 20.6% of the respondents are between 35 and 44, and 41% are over 55. Around 41.7% of the sample has completed secondary studies. Almost 53.7% have a monthly household income that ranges from €1501 to €3500. About 53.2% of the participants are of normal weight, and the majority reported no health problems. In terms of consumer segments, we found statistically significant differences between various categories for age (18–34 years and over 55 years), an education level (primary studies and university), and monthly household income (<€900–€1500). Regarding the level of education, the results suggest that individuals with secondary education were under-represented, while those with higher education were over-represented. Many studies tend to have a high proportion of university-educated participants because more educated people are more inclined to participate [27,55].

### 3.2. Purchase Habits and Attribute Importance 

The varying purchase habits and attribute importance corresponding to different consumer segments are presented in Table 3. 

Regarding purchase habits, more than half of the consumers (52.3%) state that they purchase fat-free yoghurts, followed by those that are low in sugar (44%), and ones that contain a source of calcium (31.7%). The relative attribute importance for yoghurt is highest for taste, followed by health (i.e., the product is healthy), natural ingredients, and NC and HC content. Concerning to the statistically significant differences between segments, we found differences between the fat-free labels on the purchased yoghurt and three attributes that are important to our segments when purchasing yoghurts (price, NCs, and HCs, see Table 3).

### 3.3. Nutritional Information Use and Interest in Healthy Eating 

Finally, the results from the descriptive analysis of nutritional information use and interest in healthy eating are presented in Table 4. Our findings suggest that the segments differ in terms of nutritional information use when making most food selections. Likewise, in terms of interest in healthy eating, the consumer groups differ in assigned importance to low-fat products in their diet, and whether they avoid foods that may raise cholesterol (Table 4). 

### 3.4. Utility Estimates of Latent Classes

The LC model was estimated using NLogit 6.0 Econometric Software, Inc. (http://www.limdep.com/products/nlogit/). To estimate the optimal number of segments, we constructed models with one to five classes for each product category. The model fit information criteria, such as the Akaike information criterion (AIC) and Bayesian information criterion (BIC), as well as the log-likelihood values, are normally used to discuss the relative fit with the selected number of optimal segments (Table 5). 

The lower the information criteria, the better the model fit. It is known that using BIC (AIC) tends to under-fit (over-fit) models, while evidence from previous studies [58,59] shows that AIC3 (with three weights instead of two for parameter penalisation) outperforms the other two, correcting for over-fitting effects. Nevertheless, the BIC assumes that one of the models is the true one, which is unlikely to be the case here, as the calculated information criteria continuously decreased. Previous research with similar issues [27,60] has reported that, besides the AIC and BIC, other factors that help to define the number of segments are accounting for changes in ρ-2 and lowering standard errors. Considering that the ρ-2 is normalised to the model with three segments, and the estimated parameters in the one with four and five segments started to deteriorate due to higher standard errors, we chose the LC with three segments. In other words, the estimated parameter in model four- and five-segment models started to deteriorate, resulting in larger standard errors. According to previous research, this signals the termination of model estimation with a higher number of segments [27]. (Data are available upon request) Table 6 illustrates the results of the LC model for three segments (HC-oriented, NC- and HC-claim oriented, and indifferent) and the MNL model for comparison. 

As expected, the no-buy alternative is negative and statistically significant in the MNL model, and two out of the three segments of the LC model indicate that consumers obtain higher utility from choosing any NC and/or HC product than the no-buy option. Most NCs and HCs in the MNL model are positive and statistically significant, suggesting that the utility for participants increases when these claims are present on yoghurt FOPs compared to the baseline (i.e., the unlabelled yoghurt). However, these results are not the best representation of consumer behaviour, as the log-likelihood and the AIC indicate that the LC model is superior in terms of statistical properties. The estimated parameters for the three segments suggest heterogeneity in preferences across segments.

Segment 1 (HC-oriented) represents 34.7% of respondents, segment 2 (NC- and HC-oriented) 50.4% of the respondents, and segment 3 (indifferent) 14.9%. The first group attaches higher utilities to health claims and is indifferent about NCs. More precisely, this segment mostly valued the calcium HC, which is present in the market (e.g., ‘calcium is necessary for maintaining bones under normal conditions’) followed by the one for sugar (‘consumption of food containing sweeteners instead of sugar induces a lower blood glucose’) and the calcium HC that is absent from the market (‘calcium contributes to normal muscle function’). All NCs in this segment are non-statistically significant, indicating no effect on the utility of the participants. The second group of shoppers is characterised by a high utility in terms of both NCs and HCs. Finally, indifferent consumers attach negative utilities to most NCs and HCs.

### 3.5. Characterisation of Consumers for Yoghurts with NCs and HCs 

The estimated parameters for the three segments confirm that there is heterogeneity across segments because the estimated values differ substantially between them, not only in magnitude but also in sign. The HC-oriented (S1) segment (34.7% of participants) is likely to be: female, over 55 years old, primary-educated, and in the low monthly household income bracket (i.e., <€900–€1500, see Table 2). In contrast with the other two segments (NC- and HC-oriented, indifferent), the HC-oriented group stated that free-fat information is mentioned on the FOP of the yoghurt that they habitually buy. These consumers attach the highest importance to NCs, followed by HC content, compared to the other two segments (see Table 3), and they use the nutritional information on the FOP when making most food selections. They also believe it to be important that their diet is low in fat (see Table 4). In terms of the utility attached to NCs and HCs, the respondents in this segment attach the highest utility to HCs out of all the groups, and they are indifferent towards NCs. They attach the greatest utility to HCs related to the fat content (*Hcp_fat* [3.86]), followed by sugar (*Hca_sug* [3.73]), and calcium content (*Hca_cal* [3.409], see Table 6). 

The NC- and HC-oriented segment make up 50.4% of the participants, they are more likely to be male, older than 55, with university degrees and low household income (Table 2). The consumers in this segment chose the content of an HC on the package and the price as the most important attributes when purchasing yoghurts (Table 3). They exhibit lower interest in healthy eating compared to the HC-oriented segment, and they do not avoid foods that may raise their cholesterol (Table 4). However, they attach positive utility when NCs are present along with HCs on the yoghurt packages. More specifically, these consumers attach the highest importance to nutrition information related to vitamin B_6_ content (*Hcp_vit* [1.46] and *Hca_vit* [1.16]), followed by calcium (*Hcp_cal* [1.23], Table 6). 

Lastly, the indifferent segment contains the smallest percentage of participants (14.9%). This segment consists of young female consumers between 18 and 34 years old, who have completed university studies (see Table 2). This group attaches high importance to fat-free yoghurts, believe HCs to be the most important attribute in purchasing yoghurts, and use nutritional information less frequently than the other two segments (Table 3 and Table 4). They deem it important that their diet is low in fat, but they also reported not avoiding the purchase of foods that may raise their cholesterol (Table 4). The respondents in this segment attach a much lower utility compared to NC- and HC-oriented group to claims related to the fat content of the product (*Hca_fat* [0.92]), followed by fibre (*Hcp_fib* [0.75]) and sugar (*Hca_sug* [0.51]). However, utility declines when other NCs and HCs are present on the yoghurt package (Table 6). The no-buy alternative in this segment is also non-statistically significant, indicating that consumers in this group are indifferent about the presence of NCs and HCs on yoghurt packages.

## 4. Discussion 

Overall, the results indicate that consumers positively value both NCs and HCs on yoghurt FOPs. This is consistent with the general literature review findings that consumers are willing to pay premium prices for these type of claims [15,17,20,26,27,61,62]. In addition, this result aligns with previous research, which suggests that individuals prefer dairy products with HCs and NCs rather than similar ones without these claims [18,63,64]. In this study, however, we identified three segments with heterogeneous preferences across consumers: HC-oriented (34.7% of participants), NC- and HC-oriented (50.0%), and indifferent (14.9%).

In terms of gender, our results reveal the presence of a gender dimension in the preference for yoghurts with NCs and HCs, highlighting that women (HC-oriented) display higher levels of acceptance for fat-free yoghurts and yoghurts with added calcium than men do (NC- and HC-oriented). This is consistent with Johansen et al.’s (2011) study, which found more positive attitudes towards low-fat yoghurts among Danish, Norwegian, and U.S. (Californian) female consumers compared to male shoppers [65]. In the same line, our results agree with Wardle et al. (2004), who report that women are more health-conscious than men and that the former mainly prefer fat-free or reduced-fat dairy products because they support weight control [66]. Concerning the calcium content, our results illustrate that older women perceive higher utility for calcium-related HCs (‘calcium is necessary for maintaining bones under normal conditions’ and ‘calcium contributes to normal muscle function’) present on yoghurt packages (HC-oriented). This result is consistent with the previous research [18,67] findings that female consumers are more willing to try yoghurts with added calcium. One reason that women prefer functional dairy products that are rich in calcium and promote bone health is due to their higher risk of developing osteoporosis [63,67,68].

With respect to age differences among segments, we found that HC-oriented as well as NC- and HC-oriented consumers who are older than 55 years attach higher utilities to both types of claims compared to younger members of the indifferent group (18 to 34 years old). This result agrees with previous studies, which have reported that being older is positively associated with a higher interest in dairy products that promote disease risk-reduction properties such as lowering cholesterol [63,69,70]. In addition, older consumers have been exposed for a longer period of time to food products with functional properties, hence, they are more knowledgeable and familiar with functional dairy products and their effects on health [69,70,71].

Besides age, another interesting finding is one of homogeneity: the majority of people of normal body weight across all segments evaluate taste as the most important attribute. Having a normal body weight and no health problems (Table 3) also explains the behaviour of consuming tasty food that may raise cholesterol. Hence, regarding preferences in taste, the results suggest that participants across all segments are highly sensitive to the taste of food, and they do not compromise on this aspect for the sake of health. This observation is even stronger among the participants who are indifferent towards and disinterested in purchasing yoghurts with NCs and HCs. This result is consistent with ones reported by Verbeke (2006), who found that consumers who purchase functional foods in Belgium are also not ready to compromise taste for health [72].

The results regarding NC and HC preferences suggest that, overall, consumers from all segments prefer yoghurts with these claims compared to those without. However, when it comes to comparing higher utilities between NCs versus HCs, the study demonstrates that the latter carry higher utility. In other words, presenting both types of claims together on yoghurt packages generates higher preferences. This finding differs from that of Barreiro et al. (2010b), who obtained negative utility from the combination of NCs and HCs on the package of a less healthy product (pork frankfurter sausage) [62]. However, our results are consistent with other studies that have explored consumer preferences for functional food products. Among the many claims available on the market, shoppers generally prefer HCs to NCs [63,73,74,75].

These results have practical implications for food companies and public authorities. Presenting both types of claims on the package can be used as a differentiation strategy by food companies. For the operators of the agri-food sector, the diffusion of foods with NCs and HCs can represent an opportunity to grab by means of implementing marketing strategies aimed at the different consumer segments. Policymakers will have to introduce HCs that are highly valued by consumers (e.g., *Hca_sug* and *Hca_cal*) but are not yet available on the market for yoghurts. Although the level of education is increasing and people today are more informed than ever before, there is still a segment of consumers (i.e., young people without any health problems) who are indifferent towards consuming products with NCs and HCs, and who do not avoid foods that may raise cholesterol. Hence, in terms of public health nutrition aspects (We thank an anonymous reviewer for pointing out this possibility), it may be constructive to use behavioural insights rather than device new policies. In this context it is worthwhile to introduce healthier-eating programmes and reinforce the consumption of healthy diets (e.g., the Mediterranean diet) to young Spanish people and combine it with food products with NCs and HCs. Five decades ago, the Spanish diet was a typical example of the Mediterranean diet, however, lately, Spanish consumers have moved away from that pattern [76]. Previous research, among others, the PREvention con DIeta MEDiterranea (PREDIMED) suggested that better adherence to the Mediterranean diet pattern together with a regular physical activity exerts a greater impact in lowering obesity and all-cause mortality [77,78,79,80,81]. With respect to the dairy products and precisely yoghurts, which form part of Mediterranean diet, it is well demonstrated that whole-fat and low-fat yoghurt consumption is associated with a reduced risk of general obesity [77,82] and also abdominal obesity [83,84]. Therefore, public expenditure could encourage the promotion of typical Mediterranean products with NCs and HCs in high schools and colleges. The extensive use of TV for educational purposes to reach children with an attractive food program, linking healthy food habits with sports celebrities and leisure offers, as well as to search for more accurate the appropriate combination of healthy food based on the ingredients of the Mediterranean diet is also another form of educating consumers. Finally, the popularity, acceptability, and generally perceived healthy image of yoghurt all make it an ideal snack or meal accompaniment in many cultures. The consumption of yoghurt as healthy food can be promoted especially among adolescents whose consumption of milk is low, hence, yoghurt can be considered as a milk substitute. Yoghurt should not replace fruit as a typical dessert of the Mediterranean diet but public health interventions should promote its consumption on health and educational campaigns as it plays a role in the prevention of weight gain and overweight/obesity [82]. 

Finally, this study has some limitations and further research opportunities. First, due to limited funding, it was conducted in Spain. Hence, it should be replicated in other countries to provide more evidence. Second, future research using choice experiments should be developed, not only in laboratory conditions but also in a supermarket with real products to test consumer preferences and decision making in different contexts. In our study, we used schematic choice cards as opposed to actual product packaging, which would have been more realistic (see for example [85]). In addition, hypothetical choice experiments do not use actual purchase and monetary risk, which is still a disadvantage compared to real choice experiments. Therefore, care should be taken in fully translating our results to real-life choice situations. Conducting real choice experiment with real products and real economic incentives will increase realism and avoid the hypothetical bias, which is a limitation in our research. Third, the FOP of a food product generally includes not only the NCs and HCs but also other extrinsic information (e.g., price, brand name, ingredients list, symbols, etc.). Therefore, further studies should include packages carrying other information cues in addition to NCs and HCs to evaluate the impact of these attributes in a choice environment. Finally, in terms of climate impacts (We thank an anonymous reviewer for pointing out this possibility) (e.g., greenhouse gas emissions, blue water footprint, land use etc.) associated with shifts to diets and dietary recommendations, it is important to point out that yoghurt is a dairy product, which presents a high carbon footprint per caloric intake. The previous research of Heller and Keoleian’s suggested that following a diet reduced in calories (estimations based on consumption rather than intake) results in a 1% decrease in diet-related greenhouse gas emissions [86]. In addition, Meier and Christen’s found that following an iso-Caloric shift to the German Nutrition Society Official food-based dietary recommendation could reduce energy use by 7%, blue water use by 26%, emissions by 11%, and land use by 15% [87]. Lastly, Vanham et al. (2013) determined that shifting to the same German dietary guidelines within the EU and Croatia while also accounting for a reduction in caloric intake reduces the diet-related blue water footprints by 18% [88]. Taking into account these studies, it would be very interesting to investigate in the future whether the Spanish consumer who attaches more importance to NCs and HCs on dairy products contribute or not to climate impacts such as reducing energy use, emissions, and blue water footprint. 

## 5. Conclusions

In this paper, we studied the relationship between choice behaviour, attitudes and socio-demographic characteristics and evaluated the effectiveness of consumer characteristics in predicting Spanish consumers’ choice of products with NCs and HCs. Consumers generally understand the connection between food and health, and many have an interest in the use of NCs and HCs. However, the degree of interest to use NCs and HCs differs amongst consumers and coexists with other aspects of food products (e.g., price and taste). Overall, our results suggest that there is heterogeneity in consumer preferences for multiple NCs and HCs in the Spanish marketplace. We found three segments of consumers (1—HC-oriented, 2—NC- and HC-oriented, and 3—indifferent) with regards to yoghurts carrying NCs and HCs. In addition, our findings suggest that HCs, which report the nutrient (NC) as well as the benefit of that nutrient to our health (HC), are more valued than NCs presented on the yoghurt FOP alone. Our study has contributed to drawing a clearer view of the relationships between socio-demographic and attitudinal characteristics and choice behaviours, which can be of great help in developing new products and implementing specific marketing strategies. 

## Figures and Tables

**Table 1:** Levels of nutritional and health claims and variable names used.

No.	Attributes and Levels	Variable Names ^a^	Presence ^b^ (%)
**Nutritional Claims**			
1	Fat-free	Nc_fat	(42.78)
2	Low sugars	Nc_sug	(11.99)
3	High fibre	Nc_fib	(1.09)
4	Source of vitamin B_6_	Nc_vit	(10.63)
5	Source of calcium	Nc_cal	(21.25)
6	Unlabeled (Baseline)	Nc_nat	(12.26)
**Health Claims**			
1	Reducing consumption of saturated fat contributes to the maintenance of normal blood cholesterol levels (A) *	Hca ^c^_fat	-
2	Consumption of food containing sweeteners instead of sugar induces lower blood glucose (A)	Hca_sug	-
3	Fibre contributes to an acceleration of intestinal transit	Hcp ^d^_fib	3.80
4	Fibre contributes to an increase in faecal bulk (A)	Hca_fib	-
5	With vitamin B_6_ that helps your defences and reduces fatigue	Hcp_vit	10.33
6	Vitamin B_6_ contributes to the normal functioning of the nervous system (A)	Hca_vit	-
7	Calcium is necessary for maintaining bones under normal conditions	Hcp_cal	2.17
8	Calcium contributes to normal muscle function (A)	Hca_cal	-

Notes: * indicates that a heath claim (HC) has not yet been introduced to the local market—absent (A). ^a^ Represents a variable name for the nutritional claims (NCs) used in the model estimations. ^b^ Indicates the percentage prevalence of NCs and HCs found on yoghurt packages. ^c^ Hca represents an HC that is not present in the market (absent), whereas ^d^ Hcp represents one that is.

**Table 2:** Descriptive analysis of socio-demographic characteristics in percentages, *n* = 218.

	Sample	Population	HC-Oriented	NC- and HC-Oriented	Indifferent
Sample size	218	-	34.70	50.40	14.90
**Gender ^1^**					
Male	47.25	49.02	46.05	51.35	37.50
Female	52.75	50. 98	53.95	48.65	62.50
**Age of responders ^1^**	48.8 (15.26) ^c^	42.90	-	-	-
From 18 to 34 years ***	19.72	22.24	6.67 ^a^	23.42 ^a^	37.50 ^b^
From 35 to 44 years	20.64	19.55	24.00	19.82	15.63
From 45 to 54 years	18.35	18.28	17.33	17.12	25.00
More than 55 years ***	41.28	39.93	52.00	39.64	21.88
**Education level ^2^**					
Primary studies ***	26.61	24.88	36.00	24.32	12.50
Secondary studies	41.74	47.64	34.67	47.75	37.50
University studies **	31.65	27.48	29.33	27.93	50.00
**Monthly household income**					
<900 € to 1500 € **	37.61	N/A ^e^	46.67	35.14	25.00
1501 € to 3500 €	53.67	N/A	46.67	54.95	65.63
3501 € to >4500 €	8.72	N/A	6.67	9.91	9.38
**Body mass index** ^d^					
Normal weight	53.21	N/A	43.42	57.52	62.07
Overweight	19.27	N/A	25.00	17.70	10.34
Obese	27.52	N/A	31.58	24.78	27.59
**Self-reported health problems** ^d^					
Cardiovascular diseases (heart)	6.88	N/A	5.26	9.73	0.00
High blood pressure	15.14	N/A	13.16	14.16	24.14
High blood cholesterol	23.39	N/A	23.68	23.89	20.69
Diabetes	5.96	N/A	3.95	7.08	6.90
Osteoporosis	12.84	N/A	13.16	12.39	13.79
None of the above	35.79	N/A	36.84	32.75	34.48

Note: ** and *** indicate statistical significance at the 5% and 1% levels, respectively. ^1^ Provisional data obtained (INE) on 1 January, 2017 [56]. ^2^ OCDE [57]. Superscript letters ^a,b^ indicates that the percentages vary using the χ^2^-square test. ^c^ indicates the average (and standard deviation), whereas ^d^ indicates percentages. ^e^ means ‘not available’.

**Table 3:** Purchase habits and attribute importance.

	Sample	HC- Oriented	NC- and HC-Oriented	Indifferent
**Which type of nutrient is mentioned in the yoghurt you buy? (%)**				
Source of calcium	31.65	32.89	33.63	20.69
Fat free *	52.29	60.53 ^a^	49.56	41.38 ^b^
Low sugar	44.04	46.05	44.25	37.93
High fibre	31.19	27.63	34.51	27.59
Source of vitamin B_6_	15.60	15.79	16.81	10.34
**The importance attached to attributes when buying yoghurts (average)**				
Price ***	3.53	3.62 ^a^	3.59 ^b^	3.07 ^c^
Health	4.15	4.22	4.16	3.90
Taste	4.19	4.25	4.18	4.07
Familiarity	3.27	3.37	3.19	3.28
Natural ingredients	3.97	4.08	3.95	3.79
Nutritional claim content *	3.91	4.12 ^a^	3.87	3.52^b^
Health claim content ***	3.71	3.97 ^a^	3.64 ^b^	3.31 ^c^

Notes: * and *** indicate statistical significance at the 10% and 1% levels, respectively. Superscript letters ^a–c^ indicate that group means differ for continuous variables using the Kruskal-Wallis rank test and that the percentages vary for discrete variables using the χ^2^-square test.

**Table 4:** Use of nutritional information and interest in healthy eating.

	Sample	HC- Oriented	NC- and HC-Oriented	Indifferent
**Use of Nutritional Information (Average)**				
I usually pay attention to nutritional information when I see it in an advertisement or elsewhere.	3.53	3.57	3.58	3.24
I use the nutritional information on the label when making most of my food selections. **	3.67	3.82 ^a^	3.69	3.24 ^b^
I do not spend much time in the supermarket reading nutrition information.	2.54	2.46	2.58	2.62
I read about nutritional in magazines and books.	2.91	3.03	2.90	2.62
**Interest in Healthy Eating (Average)**				
The healthiness of food has little impact on my food choices.	2.22	2.17	2.21	2.38
I am very particular about the healthiness of the foods I eat.	3.74	3.80	3.73	3.62
I eat what I like without worrying about whether it is healthy or not.	2.14	2.16	2.10	2.24
It is very important to me that my diet is low in fat. ***	3.43	3.66 ^a^	3.39	3.00 ^b^
I always follow a healthy and balanced diet.	3.42	3.43	3.42	3.38
It is important to me that my diet contains a lot of vitamins and minerals.	3.50	3.55	3.52	3.28
The healthiness of snacks makes no difference to my food choices.	1.96	1.93	1.95	2.07
I do not avoid foods even when they may raise my cholesterol. ***	2.22	1.99 ^a^	2.30 ^b^	2.55 ^c^

Notes: ** and *** indicate statistical significance at 5% and 1% levels, respectively. Superscript letters ^a–c^ indicate that group means differ for continuous variables using the Kruskal-Wallis rank test, and that the percentages vary for discrete variables using the χ^2^-square test.

**Table 5:** Comparison of information criteria.

Segments	Parameters (p)	Log Lik. (LL)	BIC	BIC/N	AIC	AIC/N	3AIC	3AIC/N	ρ^−2^
2	39	−7287.96	14,933.5	1.557	14,653.9	1.528	14,692.9	1.532	0.30
3	59	−6814.08	14,169.1	1.478	13,746.2	1.434	13,805.2	1.440	0.35
4	79	−6540.32	13,804.9	1.440	13,238.6	1.381	13,317.6	1.389	0.37
5	99	−6301.53	13,510.7	1.409	12,801.1	1.335	12,900.1	1.345	0.39

Note: Log-likelihood evaluated at zero is −8342.84.

**Table 6:** Results: LCM model (*n* = 218).

	MNL	LCM		
		HC-Oriented	NC- and HC-Oriented	Indifferent
**Variables**	*β* Coefficient (*t-ratio*)			
No-buy	−0.50 *** (−6.53)	−0.95 *** (−3.66)	−1.95 *** (−11.61)	−0.13 (−1.00)
Nc ^a^_fat	0.23 ** (2.45)	−17.24 (0.00)	0.21 * (1.77)	−0.09 (−0.36)
Nc_sug	−0.16 * (−1.69)	0.35 (1.09)	−0.30 ** (−2.56)	−0.52 ** (−2.05)
Nc_fib	0.24 *** (3.78)	−0.06 (−0.41)	0.43 *** (4.97)	−0.04 (−0.28)
Nc_vit	−0.20 *** (−3.06)	0.02 (0.12)	−0.11 (−1.27)	−0.69 *** (−3.86)
Nc_cal	−0.05 (−0.77)	0.05 (0.28)	0.06 (0.70)	−1.33 *** (−5.18)
Hca ^b^_fat	1.73 *** (18.08)	22.60 (0.00)	1.03 *** (8.66)	0.92 *** (3.85)
Hca_sug	1.10 *** (12.01)	3.73 *** (11.57)	0.26 ** (2.21)	0.51 ** (2.03)
Hcp ^c^_fib	0.92 *** (14.33)	1.46 *** (8.93)	0.96 *** (10.48)	0.75 *** (4.59)
Hca ^d^_fib	0.08 (1.09)	−0.35 * (−1.89)	0.12 (1.13)	0.50 *** (3.12)
Hcp_vit	1.61 *** (19.75)	3.40 *** (15.04)	1.46 *** (13.00)	0.28 (1.32)
Hca_vit	1.33 *** (18.21)	3.16 *** (16.33)	1.16 *** (11.50)	−0.32 (−1.58)
Hcp_cal	1.44 *** (18.63)	3.86 *** (15.44)	1.23 *** (11.73)	−0.77 *** (−2.73)
Hca_cal	1.05 *** (14.95)	3.40 *** (16.23)	0.76 *** (7.88)	−1.98 *** (−5.66)
**Segment Size**	-	34.70 *** (10.43)	50.40 *** (14.45)	14.90 *** (6.12)
N	9589	9589		
Log-lik.	−8342.84	−6814.08		
K	19	59		
AIC	1.744	1.434		

Notes: *, **, and *** indicate statistical significance at the 10%, 5%, and 1% levels, respectively. ^a^ Nc = nutritional claim; ^b^ Hc = health claim; ^c^ Hcp = health claims present in the local market; ^d^ Hca = health claims absent from the local market.

## Appendix A

**Table A1:** Population by sex and age in Spain and town (%).

Total		Sex ^a^		Age					
		Female	Male	0–14	15–34	5–54	55–64	65–84	85 and More
Spain	46,624,382	51	49	15.06	22.59	32.20	11.76	15.60	2.79
Zaragoza	1,317,847	50	50	14.06	21.13	31.53	12.24	17.24	3.80

Source: Spanish Census of Population, 2017 www.ine.es—(^a^) in percentages.
